# Mucinous Cystadenoma Arising in a Mature Cystic Teratoma in a 25-Year-Old Patient

**DOI:** 10.1155/2014/649521

**Published:** 2014-05-07

**Authors:** Michelle D. Pintea

**Affiliations:** Trinity School of Medicine, P.O. Box 885, Ratho Mill, Kingstown, Saint Vincent and the Grenadines

## Abstract

Coexistence of a mucinous cystadenoma arising in a mature cystic teratoma is infrequently reported. Herein a case of a 25-year-old woman diagnosed with a right ovarian mucinous cystadenoma arising in a mature cystic teratoma is reported. She presented with lower right abdominal discomfort. Ultrasound showed a 14.8 × 7.9 × 12.5 cm structure on the right adnexa. She underwent a diagnostic laparoscopy, which was converted to exploratory laparotomy, during which a right salpingo-oophorectomy was performed.

## 1. Introduction

Cystadenomas account for 30% of ovarian tumors in females between the ages of 20 and 45 years [1], while mature cystic teratomas account for 15–20% of germ cell tumors of the ovary. Together, 2–11% of ovarian mature cystic teratomas are associated with mucinous tumors [2].

Presented is a case of a 25-year-old female with a mucinous cystadenoma arising in a mature cystic teratoma on the right ovary.

## 2. Case Presentation

A 25-year-old nulligravida Caucasian female presented with complaints of right sided pelvic pain and sensation of fullness in her lower abdomen. On initial physical exam, a right adnexal mass could be felt. Pelvic ultrasound showed a 14.8 × 7.9 × 12.5 cm structure on the right adnexa. The structure appeared cystic, anechoic with several septations and appeared to be part of the right ovary. No pathological flow was noted and no free fluid in the Pouch of Douglas was seen. The left ovary appeared unremarkable and the uterus was noted to be within normal limits. The rest of the physical exam was unremarkable. The tumor marker CA 125 was 14.1 (normal range 0–35 U/mL) and CA 19.9 was 9 (normal range 0–37 U/mL).

The patient was admitted for a diagnostic laparoscopy, which was converted to exploratory laparotomy. A right salpingo-oophorectomy was performed. Findings included a large simple-appearing right ovarian cyst occupying the entire right ovary with no normal residual ovarian stroma. An unremarkable left ovary, uterus, appendix, and liver were noted.

The structure removed was diagnosed as a mucinous cystadenoma arising in a mature cystic teratoma measuring 15 × 11.5 × 7.5 cm and weighing 668 gm. The pathological report found the surface of the cystic mass to be smooth and intact. On the cut section, the cyst was multilocular and contained clear fluid. The inner wall of the cyst was found to be 5.5 × 2 × .5cm thick with yellow cheesy material. The fimbriae were noted to be fused. The final pelvic wash was found to be negative for malignant cells and positive for reactive mesothelium cells.

## 3. Discussion

Ovarian neoplasms are thought to have three possible origins, surface epithelial-stromal tumors, sex cord-stromal tumors, and germ cell tumors. Surface epithelial-stromal tumors are divided into serous tumors, mucinous tumors, endometrioid tumors, clear cell tumors, transitional cell tumors, and epithelial-stromal tumors. Mucinous tumors are the most common and are classified into 3 main categories, benign (cystadenoma), borderline, and malignant. Cystadenomas are the most common type of mucinous tumors [3] and comprise about 30% of ovarian tumors in females between the ages of 20 and 45 years [1]. The pathogenesis of mucinous ovarian tumors is not well understood; however, one consistent finding is the mutation of the KRAS protooncogene (58% in benign mucinous cystadenomas) [1]. One study conducted showed that KRAS mutation does occur in benign and malignant tumors, which can suggest that the mutation might be an early event in the pathogenesis of tumor progression [4]. KRAS was not tested in this patient.

The origin of teratomas is still widely disputed. The most accepted theory is that they arise from primordial germ cells [2]. Teratomas are divided into three categories, mature, immature, and monodermal [1]. Mature cystic teratomas comprise about 15–20% of germ cell tumors of the ovary [1, 5, 6]. These tumors are usually first noticed during the active reproductive years [1, 7] and they all have a karyotype of 46,XX [1, 2]. The majority (88%) of cystic teratomas are unilocular [8]. About 60% of mature cystic teratomas measure 5 to 10 cm in diameter while 10% are larger than 15 cm [7].

In conclusion, mucinous cystadenoma arising in a mature cystic teratoma is not widely reported. Studies have shown that only 2–11% of ovarian mature cystic teratomas are associated with mucinous tumors [2]. Testing for the KRAS mutation might be useful in predicting the progression of a benign tumor into a malignant one.
